# Resveratrol alleviates the interleukin-1β-induced chondrocytes injury through the NF-κB signaling pathway

**DOI:** 10.1186/s13018-020-01944-8

**Published:** 2020-09-18

**Authors:** Hong Yi, Wei Zhang, Zhi-Ming Cui, Sheng-Yu Cui, Jian-Bo Fan, Xin-Hui Zhu, Wei Liu

**Affiliations:** Department of Orthopedic, the Affiliated Hospital 2 of Nantong university & the First People’s Hospital of Nantong, No. 6 Haierxiangbei Road, Nantong City, 226001 Jiangsu Province China

**Keywords:** Osteoarthritis, Resveratrol, MMP-3, MMP-13, MMP-1, NF-κB signaling pathway

## Abstract

**Background:**

Osteoarthritis (OA) is a regular age-related disease that affects millions of people. Resveratrol (RSV) is a flavonoid with a stilbene structure with different pharmacological effects. The purpose of the experiment was to evaluate the protective role of RSV against the human OA chondrocyte injury induced by interleukin-1β (IL-1β).

**Methods:**

Chondrocytes were isolated from OA patients and identified by type II collagen, safranin O staining, and toluidine blue staining. Differentially expressed genes in chondrocytes treated RSV were identified by RNA sequencing. Kyoto encyclopedia of genes and genomes (KEGG) pathway as well as gene ontology (GO) were further conducted through Metascape online tool. A cell counting kit-8 (CCK-8) assay was applied to discover the viability of chondrocytes (6, 12, 24, and 48 μM). Many genes associated with inflammation and matrix degradation are evaluated by real-time PCR (RT-PCR) as well as western blot (WB). The mechanism of RSV for protecting IL-1β induced chondrocytes injury was further measured through immunofluorescence and WB assays.

**Results:**

A total of 845 differentially expressed genes (upregulated = 499, downregulated = 346) were found. These differentially expressed genes mainly enriched into negative regulation of catabolic process, autophagy, and cellular catabolic process, intrinsic apoptotic, apoptotic, and regulation of apoptotic signaling pathway, cellular response to abiotic stimulus, external stimuli, stress, and radiation. These differentially expressed genes were obviously enriched in NF-kB signaling pathway. RSV at the concentration of 48 μM markedly weakened the viability of the cells after 24 h of treatment (87% vs 100%, *P* < 0.05). No obvious difference was observed between the 6, 12, and 24 μM groups (106% vs 100%, 104% vs 100%, 103% vs 100%, *P* > 0.05). RSV (24 μM) also markedly depressed the levels of PGE2 and NO induced by IL-1β by 25% and 29% respectively (*P* < 0.05). Our experiment pointed out that RSV could dramatically inhibit the inflammatory response induced by IL-1β, including the MMP-13, MMP-3, and MMP-1 in human OA chondrocytes by 50%, 35%, and 33% respectively. On the other hand, RSV inhibited cyclooxygenase-2 (COX-2), matrix metalloproteinase-1 (MMP-1), MMP-3, MMP-13, and inducible nitric oxide synthase (iNOs) expression (*P* < 0.05), while increased collagen-II and aggrecan levels (*P* < 0.05). From a mechanistic perspective, RSV inhibited the degradation of IκB-α as well as the activation of nuclear factor-kappa B (NF-κB) induced by IL-1β.

**Conclusion:**

In summary, RSV regulates the signaling pathway of NF-κB, thus inhibiting inflammation and matrix degradation in chondrocytes. More studies should be focused on the treatment efficacy of RSV for OA in vivo.

## Background

Osteoarthritis (OA) is among the uppermost kinds of degenerative disorders in joints worldwide that features cartilage degradation, osteophyte formation, and joint stiffness [1]. It is estimated that about 3 million newly diagnosed OA could be presented each year [2]. The incidence of OA increases year by year as the population ages [3]. It is critically associated with significant morbidity, mortality, and increased healthcare burden in middle aged and elderly people. It is estimated that costs associated with OA exceed 2% of the gross national product in developed countries. Annual costs of 14.8 billion were estimated in the UK for patients with OA of the knee or hip. Clinical symptoms of OA include joint pain, inflammation, stiffness, deformity, and dysfunction. Articular cartilage mainly consists of extracellular matrix and chondrocytes. Currently, there are no effective approaches to prevent and treat OA, with pharmacological treatment (celecoxib and diclofenac sodium) restricted to pain relief [4]. And intra-articular infiltrations (platelet-rich plasma, hyaluronic acid, and corticosteroids) have no improvements for functional outcomes for OA patients at long-term follow-up [5]. Total knee arthroplasty is the ultimate solution to relieve pain, dysfunction, and malformation. Therefore, we need to explore an effective treatment strategy for OA [6].

A variety of cytokines, growth factors, and enzymes, such as Interleukin-1β (IL-1β) and collagenase are involved in articular cartilage degeneration. IL-1β, one of the interleukin-1 (IL-1) cytokine family, is considered the initiator of the acute inflammatory response. In particular, IL-1β and reactive oxygen species trigger a massive cell death. IL-1β is considered as the key cytokine leading to pathogenesis of OA. IL-1β is also an important mediator of inflammatory pain [7]. Increased expression of IL-1β could promote cartilage and type II collagen destruction [8]. Furthermore, IL-1β could enhance the expression of nitric oxide (NO) as well as prostaglandin E2 (PGE2) in the articular cavity, which is a representative pro-inflammatory mediator [9]. Therefore, reducing the expression of IL-1β in the articular cavity may slow down the progress of OA [10]. It is also crucial to understand the pathogenesis of OA. Studying the pathogenesis of OA is essential for identifying more therapeutic targets for OA treatment.

Traditional Chinese medicine provides an important additional option for the treatment of OA. Traditional Chinese medicines have been used to treat various kinds of diseases including OA for a long history. Ju et al. [11] found that the Huoxuezhitong capsule could ameliorate monosodium iodoacetate (MIC)-induced OA of rats through suppressing PI3K/Akt/nuclear factor-kappa B (NF-κB) pathway. Resveratrol (RSV), a polyphenol phytoalexin, is an important constituent in grape seed extract [12]. RSV has a large amount of vital pharmacological activities, for instance, anti-inflammatory, anti-diabetic, anti-oxidant and so on [13]. Tian et al. [14] suggested that RSV could inhibit tumor progression by downregulation of NLRP3 in renal cell carcinoma. Zhang et al. [15] found that RSV has a neuroprotective effect against radiation after surgically induced brain injury by reducing oxidative stress, inflammation, and apoptosis through the Nrf2/HO-1/NF-κB signaling pathway. Vicari et al. [16] conducted a research and identified that RSV could reduce inflammation-related prostate fibrosis. Some studies have demonstrated that RSV can inhibit inflammation response in OA in vivo as well as in vitro model [7]. However, the precise mechanism of RSV against OA is not fully clear. Therefore, it is necessary to examine this mechanism in detail. With the development of RNA sequencing technology, the gene regulatory networks can be better illustrated.

Transcription factor, nuclear factor kappa B (NF-κB), is linked to cell survival, as well as inflammation response. The signaling pathway of nuclear factor-kappa B (NF-κB) is crucial for OA occurrence and progression [17]. NF-κB is a common transcription factor and plays an important role in regulating cellular responses. It also revealed that NF-κB is a crucial transcriptional factor involved in the regulation of immune and inflammation response. NF-κB activation was always accompanied with an inflammatory response. In normal condition, NF-κB bounded with IκB to formulate a complex and located in the cytoplasm [18]. IL-1β stimulation of chondrocytes leads to the degradation and phosphorylation of IκB, and following NF-κB p65 dissociates from IκB. Therefore, in the OA progression, NF-κB signaling pathway was significantly activated. Here, we performed RNA sequencing technology and revealed that RSV could significantly affect the NF-κB signaling pathway and thus delay OA progression.

We conducted this experiment to evaluate the effects, in human chondrocytes, of RSV in relieving the inflammatory response induced by IL-1β. Then, we applied RNA sequencing technology to perform the genes expressed differentially between RSV and controls. The regulation of RSV on inflammatory responses in the OA cell model was further discussed. Therefore, in this study, we hypothesize that (i) the chondrocytes isolated from articular cartilage and then co-incubated with IL-1β could induce an articular chondrocytes degradation and inflammation model; (ii) RSV has a chondroprotective effect against OA in IL-1β-induced chondrocytes inflammation; and (iii) the underlying molecular mechanisms of RSV’s chondroprotective effects might be related to the regulation of the NF-κB signaling pathway. This study provides a reference for the clinical application of RSV in OA therapy.

## Methods

### Human chondrocyte isolation

We isolated chondrocytes from articular cartilage harvested aseptically from the knee joints of OA patients. The Ethics Committee of Soochow University Second Affiliated Hospital approved this study (approval number: YLS-NFPS-2019-056), and we obtained informed consent from patients and their family members before the study. Briefly, cartilage tissue was rinsed with PBS and then segmented into small pieces (< 2 mm^3^). Then, we digested these small pieces by collagenase type II (2 mg/mL) at 37 °C for 30 min. After filtration through a 100-μm sieve, centrifuged at 175 × g for 5 min. Chondrocytes were cultured in alpha-MEM medium (α-MEM) supplemented with 20% FBS. Cell identification was facilitated by staining of collagen type II, toluidine blue O (TBO), and safranin O staining.

### Cell viability

After specific treatment (control or RSV at different concentrations), we cultured 1 × 10^4^ chondrocytes/well all together in 96-well plates and cultivated in normal conditions. Then, we added 10 μL CCK-8 to the culture media of each well. All of the wells were incubated for 2 h. Finally, the OD values were evaluated at 490 nm.

### Transcriptome sequencing

We isolated total RNA from chondrocytes by the TRIzol method in RSV and control groups. DNA was digested with DNase I (Thermo Fisher Scientific) and finally, double-stranded cDNA was produced. The double-stranded cDNA purified were end-repaired, added bases A, and then connected with sequencing adaptors. Ultimately, we checked the cDNA libraries with an Agilent 2100 Bioanalyzer and ABI StepOnePlus Real-time PCR system and subjected the cDNA libraries to RNA-sequencing using Illumina HiSeqTM 2000.

### KEGG and GO pathway analysis

To explore the functions of mRNAs expressed significantly differentially and the corresponding signal pathways, enrichment analysis of KEGG and GO pathway was conducted. First, genes expressed differentially between RSV and control groups were submitted to Metascape online tool (http://metascape.org/gp/index.html).

### Protein-protein interaction

To further analyze the potential function of these differentially expressed genes, protein-protein interaction was performed through STRING database (version 11.0) (https://string-db.org/). Subsequently, a module analysis of the network was performed using the Mcode plugin [19]. The selection MCODE criteria were as follows: max depth = 100, node score cutoff = 0.2, K − core = 2, MCODE scores ≥ 5, and degree cutoff = 2 [20].

### qRT-PCR

We separated the total RNA from chondrocytes with TRIzol reagent and then reversely transcribed into cDNAs by PrimeScript™ RT reagent Kit (Qiagen) referring to the manufacturer’s manual. Subsequently, we analyzed cDNAs via performing qPCR with Maxima SYBR Green/ROX qPCR Master mix (Takara, Japan). After that, we performed qRT-PCR with SYBR Green Premix Ex Taq kit (Takara) on MxPro Mx3005P system (Agilent Technologies, USA). The cycling qualifications of mRNAs were as follows: 95 °C for 30 s, then 40 cycles of 95 °C for 5 s, then 55 °C for 30 s, and then 72 °C for 1 min. The sequences of primer for PCR were listed in Table 1. We applied the 2^−ΔΔCt^ method to detect the expression of target genes.

**Table 1:** Primers used in this study

Gene	Forward primer	Reverse primer
MMP-1	5′-GGGAATAAGTACTGGGCTGTTCAG-3′	5′-CCTCAGAAAGAGCAGCATCGATATG-3′
MMP-3	5′-CTGGCCTGCTGGCTCATGCTT-3′	5′-GCAGGGTCCTTGGAGTGGTCA-3′
MMP-13	5′-CCAGAACTTCCCAACCAT-3′	5′-ACCCTCCATAATGTCATACC-3′
GAPDH	5′- AAGGCCATCACCATCTTCCA-3′	5′-GGA TGCGTT GCTGACAATCT-3′

### Western blot analysis

The total cellar proteins were separated applying RIPA buffer (Invitrogen, USA) in accordance with manufacture instructions. Then, we evaluated the concentration of proteins by a BCA protein assay kit. We separated 25 μg of each protein samples on 12% SDS-PAGE gels, and then transferred onto the PVDF membranes (BioRad, USA), incubated with 5% no-fat milk, cultured in the primary antibodies against PEG (Abcam, USA, 1:100), COX-2 (1:500), iNOS (1:1000), MMP-1 (1:1000), MMP-3 (1:1000), MMP-13 (Abcam, 1:1000), p-p65 (Cell Signaling Technology, USA, 1:1000), p65 (1:1000), IkB (1:1000), p-IkB (Cell Signaling Technology, 1:1000), and GAPDH (Proteintech, Wuhan, China, 1:5000) overnight at 4 °C, and then blocked at room temperature with secondary antibodies for 2 h. Finally, we used ECL detection system reagents (Millipore, MA, USA) to visualize the protein blots.

### Immunofluorescence

Chondrocytes were seeded into 20-mm confocal Petri dishes and cultured for 24 h. Then, we randomly divided the chondrocytes into three groups: IL-1β + RSV, IL-1β, and the control groups. At room temperature, we fixed the chondrocytes in each group for 15 min with 4% paraformaldehyde in PBS and then treated with 0.5% Triton X-100 (Bio-Rad, USA) for 15 min. At 37 °C, we blocked the nonspecific binding sites using 1% bovine serum albumin (abbreviated as BSA) for 30 min. Subsequently, at 4 °C, we incubated the samples in the rabbit anti-human collagen type II (COL-II) antibody (Abcam, Britain; 1:200) overnight. At room temperature, after rewarming, we incubated the samples for 30 min with FITC-labeled goat anti-rabbit secondary antibody (Boster, China; 1:800). Finally, at room temperature, we stained the nuclei with DAPI in darkness for 5 min, after washing with PBS for five times.

### Statistical analysis

The study was conducted in triple independently and values were presented as the mean ± standard deviation (SD). Statistical analyses were performed using the SPSS 20.0 statistical software. Comparisons between groups were conducted by Student’s *t* test or ANOVA with Tukey’s post hoc test. *P* < 0.05 was defined as statistically significant.

## Results

### Identification of chondrocyte

**Fig. 1:**
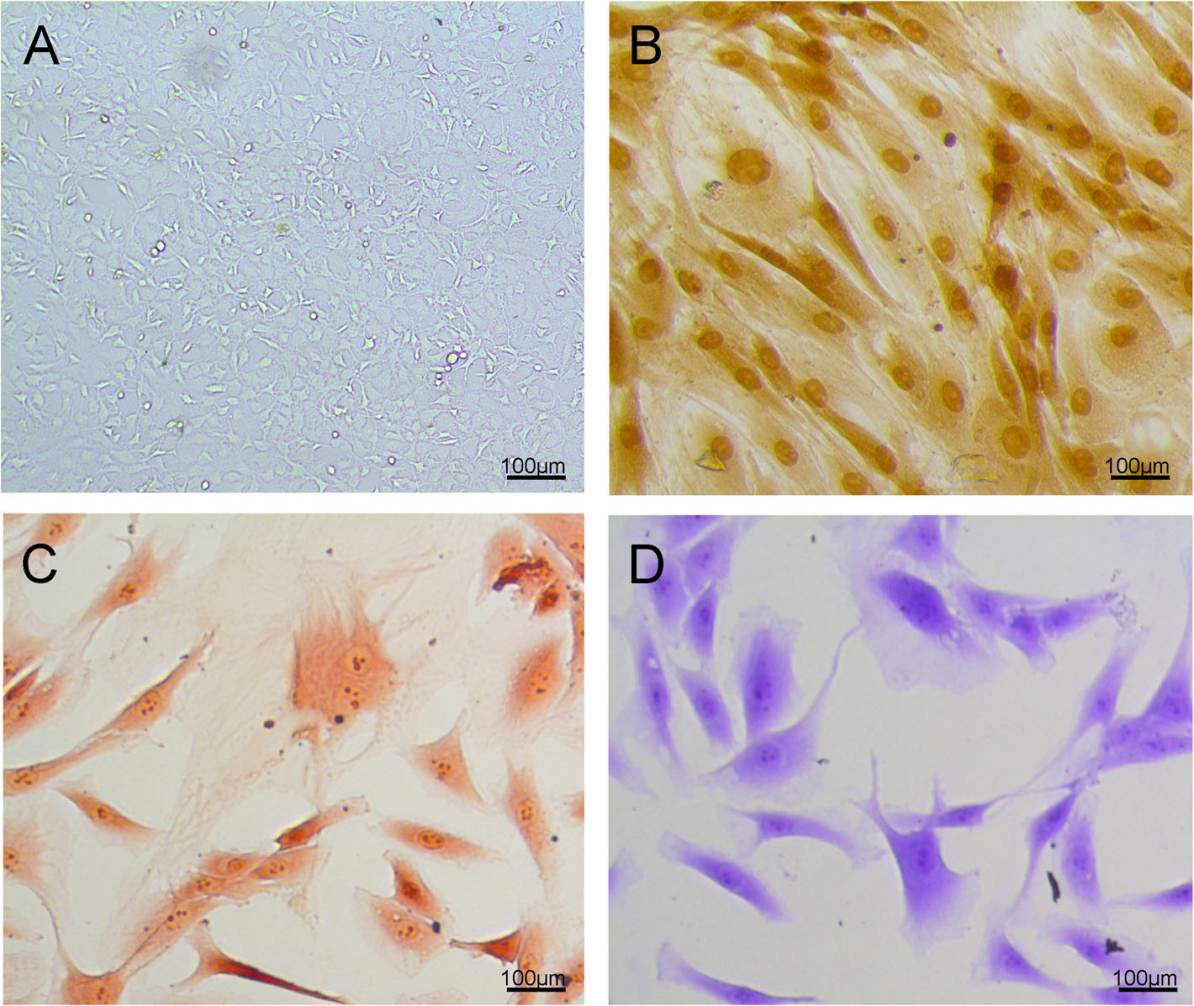
Identification of chondrocytes. (**a**) morphological of chondrocytes. (**b**) The section was stained by immunohistochemistry of type II collagen. Chondrocytes as assessed by Safranin-O (**c**) and Alcian blue (**d**) staining for acidic sulfate mucosubstances

After 7 days of culture, the P3 adherent cells were mainly fusiform, arranged regularly, and grew evenly (Fig. 1a). The cytoplasmic granules of the cells exhibited brownish yellow after immunohistochemical staining by type II collagen (Fig. 1b). Meanwhile, chondrocytes were also identified by the presence of safranin O staining (Fig. 1c) as well as toluidine blue staining (Fig. 1d).

### Differentially expressed mRNAs in chondrocytes treated with RSV

**Fig. 2:**
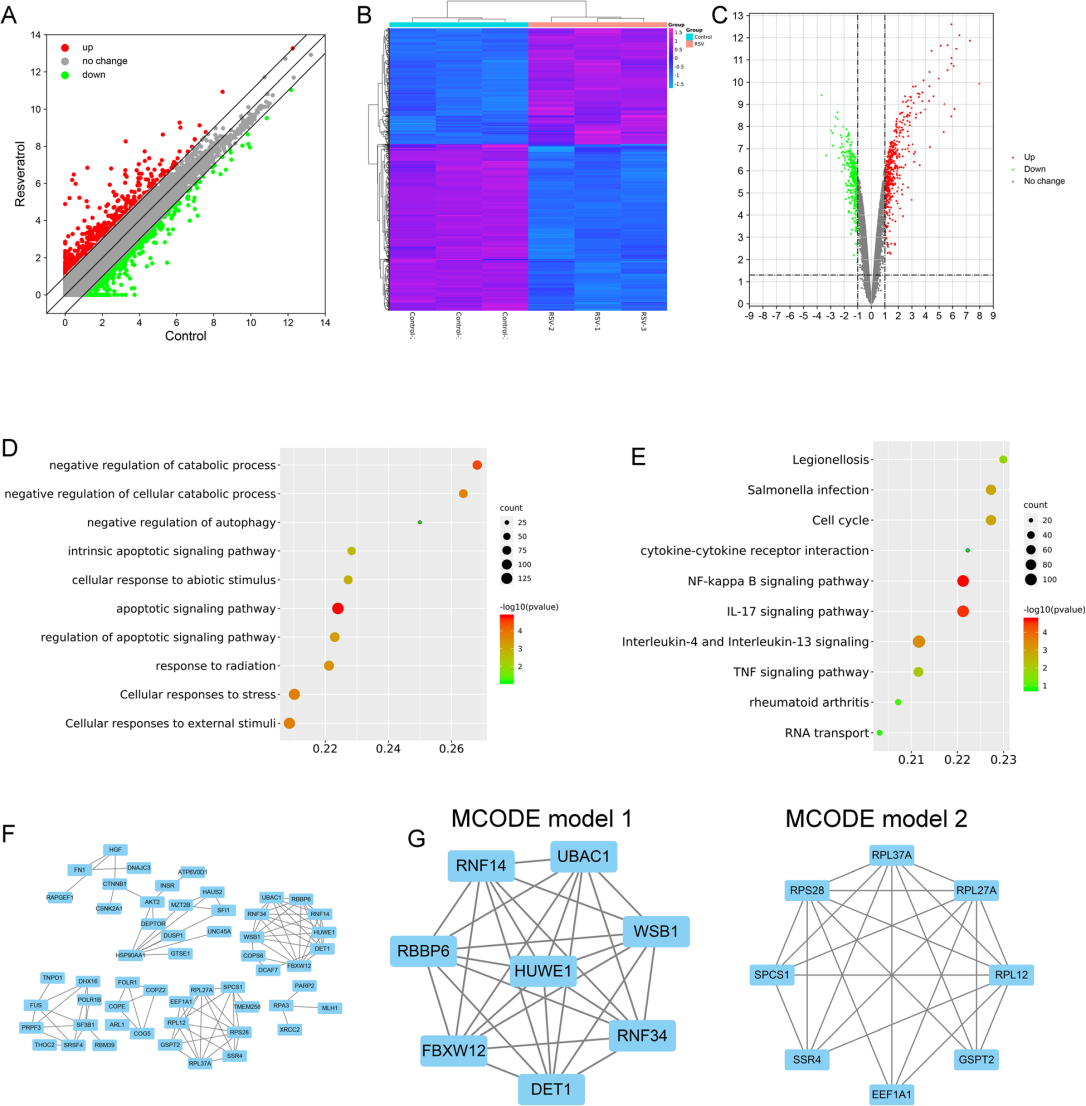
Genes expressed differentially in chondrocytes treated with or without resveratrol (RSV). **a** Scatter plot of the genes expressed differentially. Red dots referred to upregulated genes, green dots referred to downregulated genes, gray dots referred to no change. **b** Cluster heatmap of the genes expressed differentially in chondrocytes treated with/without RSV. Blue referred to downregulated genes and purple referred to upregulated genes. **c** Volcano plot of the genes expressed differentially in RSV-treated chondrocytes. Red dots referred to upregulated genes, green dots referred to downregulated genes and gray dots referred to no change. **d** Gene ontology analysis of the genes expressed differentially. The conversion of color from green to red indicates the -log10 (*P* value) expression level from the low to high. The size of circles indicates gene counts, with larger circles representing larger gene counts. **e** KEGG pathway analysis of the genes expressed differentially. The change of color from green to red indicates the -log10 (*P* value) expression value from the low to high. The size of circles indicates gene counts, with larger circles representing larger gene counts. **f** Protein-protein interaction of the genes expressed differentially in chondrocytes treated resveratrol. **g** Top 2 MCODE models in differentially expressed genes in chondrocytes treated RSV

To explore the effects of RSV on the transcriptome of chondrocytes, RNA sequencing was performed. Finally, a total of 845 differentially expressed genes (upregulated = 499, downregulated = 346) were found. A scatter plot of the 845 differentially expressed genes was obtained after standardized and log2-transformed of the raw chip data (Fig. 2a). Differential genes obtained from the RNA sequencing were visualized using heatmap (Fig. 2b) and volcano plot (Fig. 2c). A total of 845 differentially expressed genes (upregulated = 499, downregulated = 346) were found. We then used Gene Ontology (GO) analysis to gain a more comprehensive understanding of the RSV-induced DEGs. the top ten enriched GO terms (negative regulation of catabolic process, autophagy, and cellular catabolic process, intrinsic apoptotic, apoptotic, and regulation of apoptotic signaling pathway, cellular response to abiotic stimulus, external stimuli, stress, and radiation) were shown in Fig. 2d. The top ten enriched terms KEGG pathway was shown in Fig. 2e (Legionellosis, Salmonella infection, cell cycle, cytokine-cytokine receptor interaction, NF-kappa B signaling pathway, IL-17 signaling pathway, interleukin-4 and interleukin-13 signaling, TNF signaling, rheumatoid arthritis, and RNA transport). The most significantly enriched pathway was NF-kappa B signaling pathway. The protein-protein interaction (PPI) network is important to reveal the functions of proteins. PPI results generate 330 nodes and 689 edges (Fig. 2f). The entire PPI network was analyzed using the MCODE plug-in, and the top two modules were chosen (Fig. 2g).

### RSV affects human OA chondrocyte viability

**Fig. 3:**
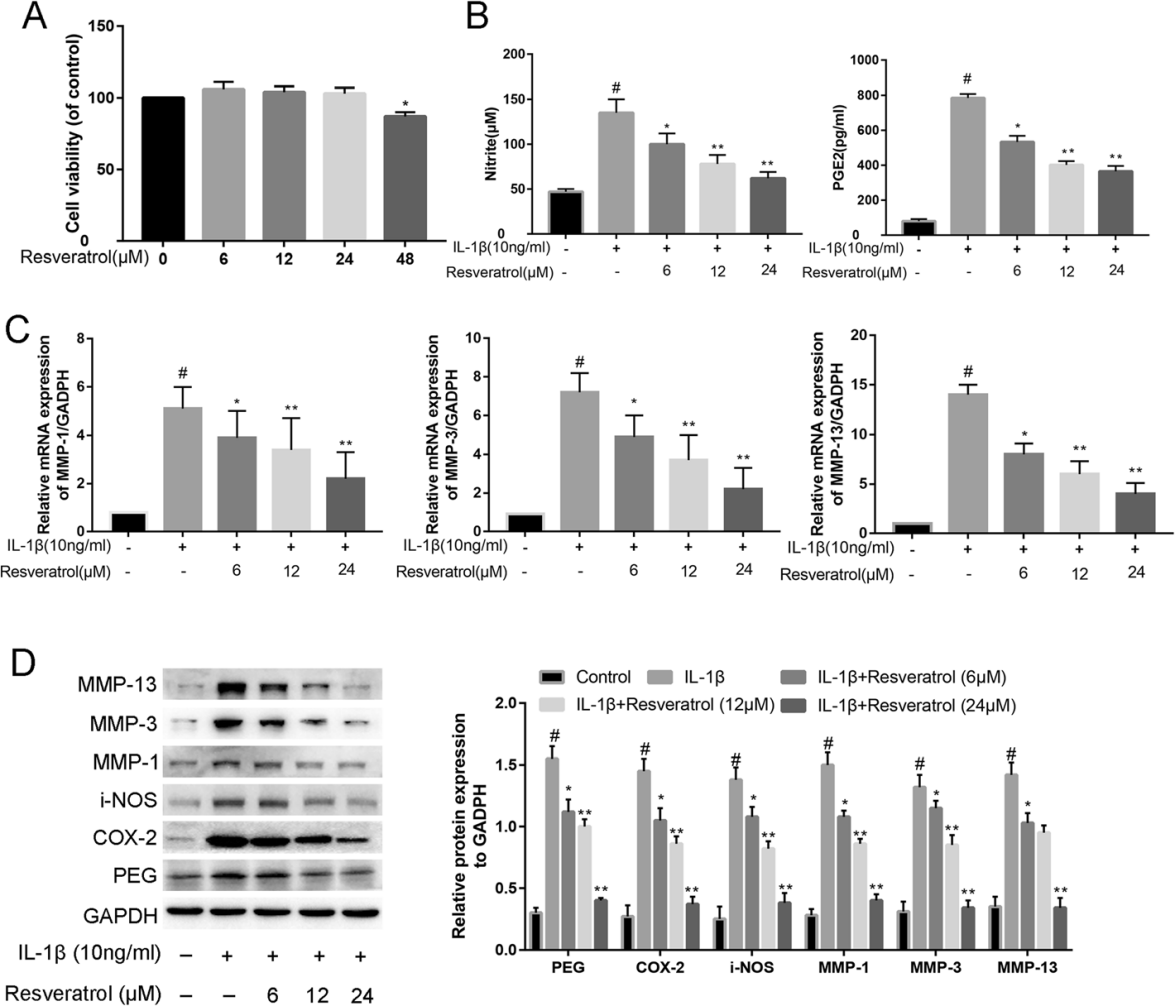
Resveratrol affects the proliferation and matrix degradation in chondrocytes. **a** Cell viabilities were evaluated by the CCK-8 assay in chondrocytes treated with resveratrol (RSV). **b** RSV depresses the production of nitrite and PGE-2 induced by IL-1β. **c** Mean relative mRNA levels of MMP-13, MMP-9, MMP-3, and MMP-1 in various groups. **d** Western blot assay was applied to assess the expression levels of PEG, COX-2, i-NOS, MMP-13, MMP-3, and MMP-1

To determine the biologically safe concentrations of RSV, chondrocytes were exposed to RSV with increasing concentrations from 6 to 48 μM for 24 h. Cell viability was analyzed using a CCK-8 assay (Fig. 3a). There was a toxic effect on chondrocytes at the concentrations of RSV exceeding 48 Μm (87% vs 100%, *P* < 0.05). No obvious difference was observed between the 6, 12, and 24 μM groups compared to that of the control group.

### Influence of RSV on nitrite and PGE2 in human OA chondrocytes

Figure 3b shows that IL-1β stimulation increases the nitrite and PGE2 production compared with unstimulated cells. RSV significantly attenuated the production of IL-1β-induced PGE2 and nitrite in a manner of concentration dependent (6-24 μM) at 24 h. RSV (24 μM) also markedly depressed the levels of PGE2 and NO induced by IL-1β by 25% and 29% respectively.

### Effects of RSV on MMP-13, MMP-3, and MMP-1 expression

As demonstrated in Fig. 3c, IL-1β dramatically increased the mRNA expressions of MMP-13, MMP-3, and MMP-1 at 24 h compared with those of the control group. Our experiment pointed out that RSV could dramatically inhibit the inflammatory response induced by IL-1β, including the MMP-13, MMP-3, and MMP-1 in human OA chondrocytes by 50%, 35%, and 33% respectively. Further, these results were confirmed by the western blot analysis (Fig. 3d). Western blot results further confirmed that RSV inhibited the protein expression of i-NOS, COX-2, and PEG (*P* < 0.05, Fig. 3d).

### Influence of RSV on the expression of COL-II

**Fig. 4:**
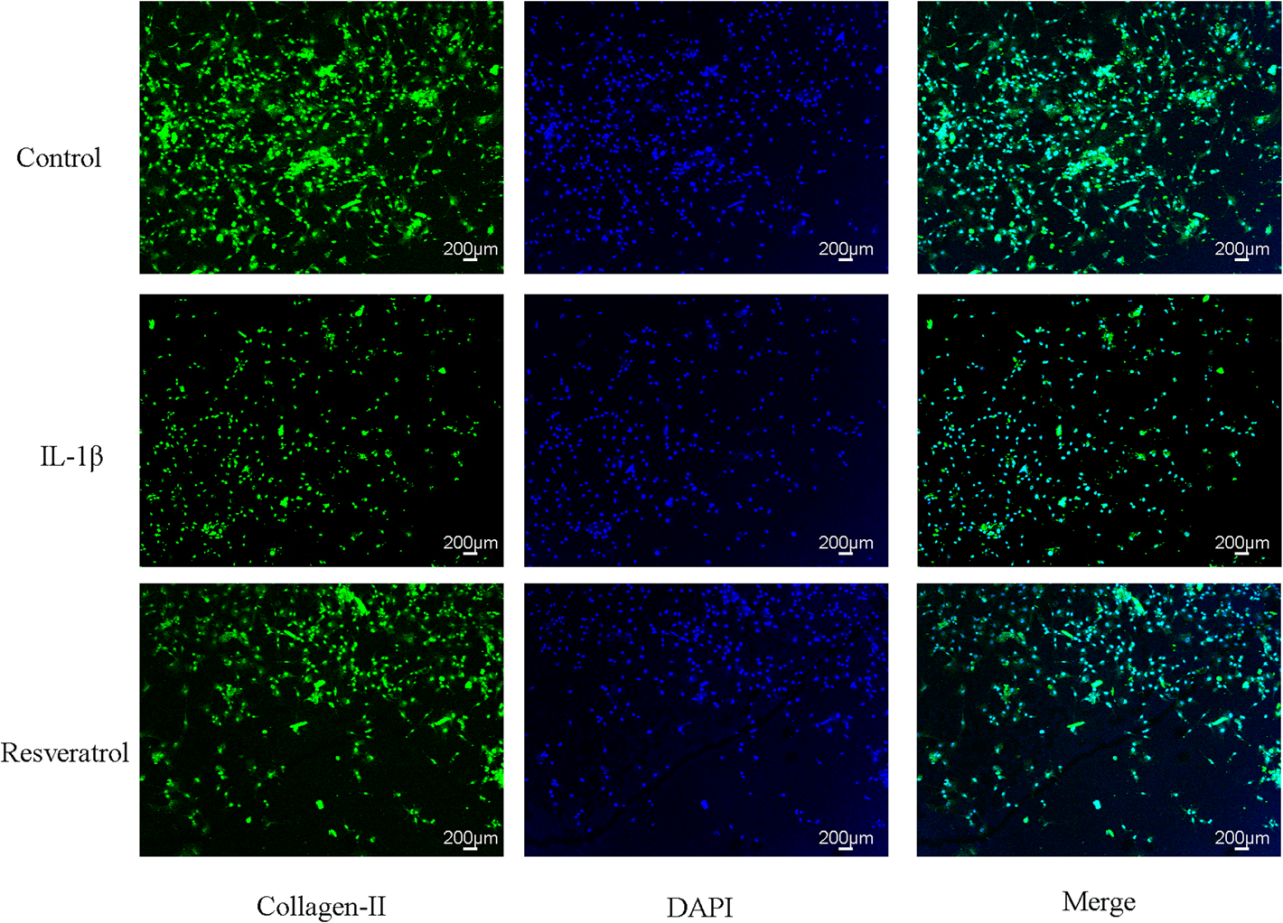
Influence of resveratrol (RSV) on collagen-II expression in human OA chondrocytes induced by IL-1β

The results of immunofluorescence were demonstrated in Fig. 4. Collagen-II expression level was markedly decreased by IL-1β. And collagen-II expression was obviously increased in the RSV group in comparison to the IL-1β group.

### Influence of RSV on NF-kB signaling pathway

**Fig. 5:**
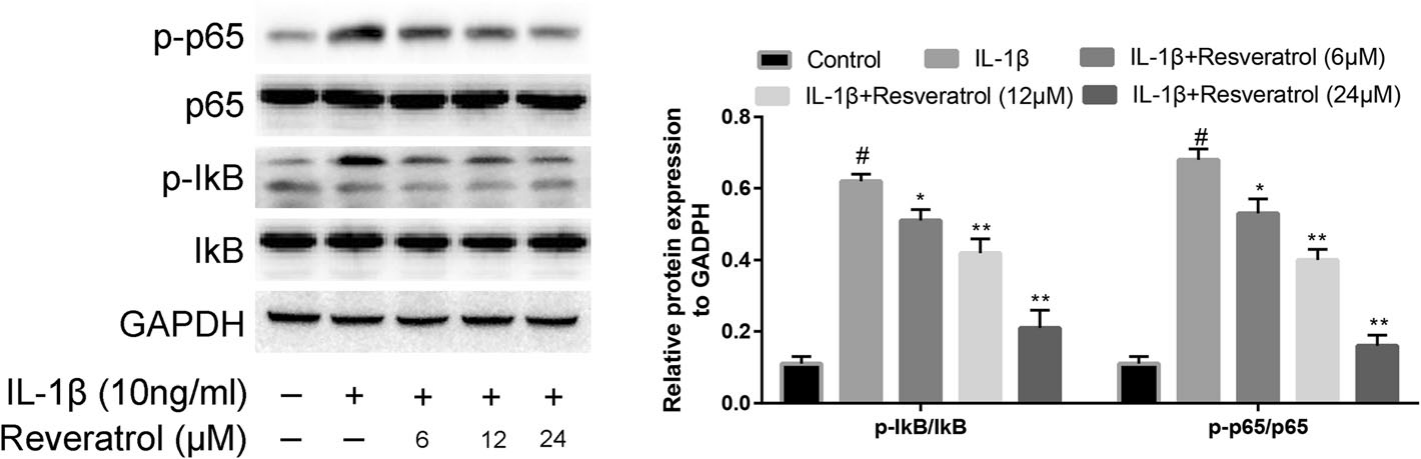
Influence of resveratrol (RSV) on the signaling pathway of NF-κB in human OA chondrocytes

As demonstrated in Fig. 5, the phosphorylation level of p65 and p-IkB was raised in chondrocytes with the IL-1β treatment by 50% and 40% respectively. Treatment with RSV resulted in reduced levels of p-IkB and p65 at 24 h by 30% and 28% respectively (*P* < 0.05). These results demonstrate that RSV regulates the inflammation response induced by IL-1β via regulating NF-kB signaling pathway.

## Discussion

RNA sequencing was further identified, the differentially expressed genes between RSV and control groups. Bioinformatic analysis found that RSV primarily regulated NF-κB signaling pathway. And RSV significantly decreased the inflammation response induced by IL-1β. The inhibition effects of RSV on inflammation response induced by IL-1β were regulated by the activation of NF-κB pathway.

RSV was first separated from a Japanese man in 1940 [21]. RSV is a nonflavonoid polyphenolic compound belonging to the stilbene group able. RSV makes plants more resistant to pathogens and improves environmental deterioration [13]. Previously, Wei et al. [13] performed an in vivo study and found that RSV could protect cartilage degradation in OA model. Xu et al. [12] also found such protective effects of RSV against inflammation in the experimental model of obesity-related osteoarthritis. Clinical trials have shown that the RSV is superior than meloxicam in pain relieving and functional recovery of OA patients [22]. However, another randomized double-blind, placebo-controlled pilot study suggested that RSV has no effect on inflammatory response after a marathon in male athletes [21]. The anti-inflammatory mechanisms of RSV have not been well defined.

Firstly, CCK-8 assay was conducted to investigate the appropriate dosage and used for further study. Dose of RSV between 6 and 24 μM was used for further investigation according to the CCK-8 assay. Another former experiment completed by Kang et al. [23] also identified that RSV had no obvious cytotoxicity even at a high concentration of 100 μM in chondrocytes.

Articular chondrocyte-generated IL-1β stimulates the development of OA. IL-1β markedly upregulate MMPs expression in chondrocytes, which contribute to cartilage destruction and finally can cause OA [24]. IL-1β with 10 ng/ml has been extensively applied to mimic OA in in vitro experiments [25]. MMPs are proteolytic enzymes that contribute to tissue remodeling. After stimulated with IL-1β, MMPs were significantly upregulated [26]. Gu et al. [27] presented that RSV suppresses the expression of IL-6 and MMP-13 in IL-1β-induced human articular chondrocytes via signaling cascades that are independent and dependent of TLR4/MyD88. As a result, we hypothesized that RSV reduces MMP production and activation during the progression of OA, then exerting an anti-inflammatory influence. Jing et al. [28] found that RSV downregulates steatosis through estrogen receptor α-mediated pathway in LO_2_ cells.

RNA sequencing results suggested that RSV mainly regulating cellular apoptotic signaling pathway and NF-κB signaling pathway. NF-κB signaling plays a central part in the regulation of inflammation as well as pathogenesis of OA. Rigoglou et al. [29] suggested that intervene with the signaling pathway of NF-κB could inhibit or delay the progression of OA. On the contrary, NF-κB signaling overactivation could enhance the inflammation response and matrix degradation [30, 31]. In non-degraded cartilage, NF-κB is in the cytoplasm, and the combination of IκB proteins with NF-κB is able to inhibit its nuclear localization and the activation of signals. With the phosphorylation and degradation of IκB, genes related to inflammatory response was increased [32]. In this study, we revealed that RSV could inhibit the phosphorylation of p65 and IkB and thus alleviate the inflammation response.

We also acknowledge that the current research has limitations. First, the antagonists of NF-κB were not administrated to identify the inflammation response after block with the NF-κB signaling pathway. Second, additional in vivo experiments (cartilage defect or OA model) are needed to verify the in vitro results.

## Conclusion

In summary, this experiment found that, in human OA chondrocytes, RSV attenuated the inflammatory response induced by IL-1β through regulation of the NF-kB signaling pathway. In the present study, we first provide evidence showing that RSV is a new therapeutic approach to prevent inflammation response in OA progression. Additional in vivo experiments are needed to verify the RSV in preventing OA progression.

## Data Availability

We state that the data will not be shared since all the raw data are present in the figures included in the article.

## Abbreviations

OA: Osteoarthritis
RSV: Resveratrol
IL-1β: Interleukin-1β
KEGG: Kyoto encyclopedia of genes and genomes
GO: Gene ontology
CCK-8: Cell counting kit-8;
RT-PCR: Real-time PCR
WB: Western blot
COX-2: Cyclooxygenase-2
MMP-1: Matrix metalloproteinase-1
iNOs: Inducible nitric oxide synthase
NF-κB: Nuclear factor-kappa B
PGE2: Prostaglandin E2
α-MEM: alpha-MEM medium
TBO: Toluidine blue O
OD: Optical density
SD: Standard deviation
COL-II: Collagen type II
PPI: Protein-protein interaction
MCODE: Molecular complex detection
